# Childhood immunisation timeliness and vaccine confidence by health information source, maternal, socioeconomic, and geographic characteristics in Albania

**DOI:** 10.1186/s12889-021-11724-6

**Published:** 2021-09-22

**Authors:** Daniela Mayerová, Kaja Abbas

**Affiliations:** Faculty of Epidemiology and Population Health, London School of Hygiene & Tropical Medicine, London, WC1E 7HT UK

**Keywords:** Immunisation timeliness, Vaccine confidence, Demographic and health survey, Health information source, Albania

## Abstract

**Background:**

Albania is facing decreasing childhood immunisation coverage and delay in timeliness of vaccination despite a growing economy and universal health insurance. Our aim is to estimate childhood immunisation timeliness and vaccine confidence associated with health information source, maternal, socioeconomic, and geographic characteristics in Albania.

**Methods:**

We used the 2017–2018 Albania Demographic and Health Survey to analyse childhood immunisation timeliness and vaccine confidence among 2113 and 1795 mothers of under-5-year-old children respectively using simple and multivariable logistic regression.

**Results:**

Among mothers of under-5-year-old children in Albania, 78.1% [95% CI: 74.3, 81.5] never postponed or rejected childhood vaccines. Immunisation delay was reported by 21.3% [18.0, 25.1] of mothers, but a majority (67.0%) were caused by the infant’s sickness at the time of vaccination, while a minority (6.1%) due to mothers’ concerns about vaccine safety and side effects. Vaccine confidence was high among the mothers at 92.9% [91.0, 94.4] with similar geographical patterns to immunisation timeliness. Among 1.3% of mothers who ever refused vaccination of their children, the main concerns were about vaccine safety (47.8%) and side effects (23.1%). With respect to childhood immunisation timeliness, after controlling for other background characteristics, mothers whose main health information source was the Internet/social media had 34% (adjusted odds-ratio AOR = 0.66 [0.47, 0.94], *p* = 0.020) lower odds in comparison to other sources, working mothers had 35% (AOR = 0.65 [0.47, 0.91], *p* = 0.013) lower odds in comparison to non-working mothers, mothers with no education had 86% (AOR = 0.14 [0.03, 0.67], *p* = 0.014) lower odds compared to those who completed higher education, and mothers living in AL02-Qender and AL03-Jug regions had 62% (AOR = 0.38 [0.23, 0.63], *p* < 0.0001) and 64% (AOR = 0.36 [0.24, 0.53], *p* < 0.0001) lower odds respectively in comparison to those residing in AL01-Veri region (*p* < 0.0001).   With respect to vaccine confidence, mothers whose main health information source was the Internet/social media had 56% (AOR = 0.44 [0.27, 0.73], *p* = 0.002) lower odds in comparison to other sources, single mothers had 92% (AOR = 0.08 [0.01, 0.65], *p* = 0.019) lower odds compared to those married/living with a partner, mothers of specific ethnicites (like Roma) had 61% (AOR = 0.39 [0.15, 0.97], *p* = 0.042) lower odds in comparison to mothers of Albanian ethnicity, and mothers living in AL03-Jug region had 67% (AOR = 0.33 [0.19, 0.59], *p* ≤ 0.0001) lower odds compared to mothers residing in AL01-Veri region.

**Conclusions:**

Reinforcement of scientific evidence-based online communication about childhood immunisation in combination with tracking and analysis of vaccine hesitancy sentiment and anti-vaccination movements on the Internet/social media would be beneficial in improving immunisation timeliness and vaccine confidence in Albania. Since parents tend to search online for information that would confirm their original beliefs, traditional ways of promoting vaccination by healthcare professionals who enjoy confidence as trusted sources of health information should be sustained and strengthened to target the inequities in childhood immunisation timelines and vaccine confidence in Albania.

## Introduction

Immunisation is a highly cost-effective public health intervention while also improving health equity locally and globally [1, 2]. However, maintaining a high level of public confidence in vaccines and immunisation programmes, and minimizing the delay and rejection of vaccination are increasingly challenging worldwide [3–5].

Immunisation timeliness refers to adherence to vaccination schedules but the recommended national immunisation schedules vary between countries and there are no commonly accepted standards for timeliness among the countries [6]. Delays in immunisation uptake may result in lack of protection during high-risk periods and failure to complete further recommended vaccinations [7].

Vaccine confidence refers to the trust in a multitude of factors – the effectiveness and safety of vaccines, the reliability of the health service delivery system, the competencies of the health professions, and the motivation of the policymakers in deciding the required vaccines [8]. Vaccine confidence influences vaccine hesitancy which refers to the delay in acceptance or refusal of vaccines despite availability of vaccine services [8].

Vaccine confidence impacts vaccination demand and varies widely among countries [4, 5, 9]. A survey on attitudes towards immunisation in 67 countries revealed that skepticism about vaccine importance and safety is a sensitive and important issue in Europe in comparison to other World Health Organization (WHO) regions [4]. In a related survey conducted in 18 European countries on the attitudes and behaviors among parents regarding their children’s immunisation, 20% of parents delayed vaccination and 12% refused vaccination while 24% of parents identified themselves as somewhat hesitant and 4% as very hesitant [9].

The midterm report of the WHO European Vaccine Action Plan for 2015–2020 reported that decreasing vaccine confidence was contributing to suboptimal immunisation coverage and delays in uptake [10]. The goal of reaching 95% or higher coverage for DTP3 (third dose of diphtheria, tetanus, pertussis) vaccination in 90% of member states was at risk, and elimination of measles and rubella transmission goals for 2015 were not met. There were 82,596 measles cases during the 2018 outbreak affecting 47 of 53 WHO European region countries, which were 3-times more than in 2017 and 15-times more than in 2016 [11]. Albania, with a population of 2.9 million people, reported 1469 measles cases during the 2018 outbreak [12].

### Childhood immunisation in Albania

Mandatory vaccination of infants aged 12–23 months in Albania includes BCG (Bacillus Calmette-Guérin) and hepatitis B vaccines at birth, three doses of DPT-HepB-Hib (diphtheria, pertussis, tetanus, hepatitis B, *Haemophilus influenzae* type b) vaccine at 2, 4, and 6 months, three doses of polio vaccine at 2, 4, 6 months, three doses of PCV (pneumococcal) vaccine at 2, 4, and 10 months, and one dose of MMR (measles-mumps-rubella) at 12 months. For infants 24–35 months old, additional mandatory immunisation consists of booster doses of DTP and polio at 2 years [13]. A booster dose of measles vaccine is administered at 5 years [13] and is not considered in this study.

Albania is facing decreasing childhood immunisation coverage despite a growing economy and universal health insurance [14], rising expenditure in health services [13], and free of charge childhood immunisation [15]. The proportion of zero dose children (children who never received any vaccines) was below 1% in 2017–18 [14] as it was in 2008–2009 [16]. However, basic vaccination coverage (BCG, three doses of DPT-HepB-Hib, three doses of polio vaccine, and one dose of measles-containing vaccine) in 12–23 months old children declined from 94% in 2008–2009 to 75% in 2017–2018 [14]. While in 2017–2018, 75.0% of 12–23 months old children and 87.9% of 24–35 months old children received all basic vaccinations, rates of all age-appropriate vaccines were 67.2% in 12–23 months old children and 74.5% in 24–35 months old children [14]. These differences indicate immunisation delays.

### Health information source, maternal, child, socioeconomic, and geographic determinants of immunisation timeliness and vaccine confidence

The Internet is a valuable source of information related to vaccines and health in general but also spreads substantial misinformation regarding immunisation [17–19]. People seeking information on vaccines on the Internet find mostly pro-vaccination documents [19–23]. However, parents searching online about childhood vaccines tend to have more negative attitudes towards immunisation compared to parents with other sources of health-related information [24–27].

A recent systematic review described child gender, maternal education and age, socioeconomic status, urban/rural residence, household size, ethnicity, birth setting, child age, birth order, maternal occupation, marital status, paternal education, distance to clinic, antenatal care visits, maternal and parental occupation, birth year, religion, region, and paternal age as the most commonly tested predictors of childhood immunisation timeliness in low- and middle-income countries [6]. Though Albanian children’s immunisation pattern is similar to that in high-income countries, having achieved high vaccination uptake followed by a decline over the last decade, social determinants of health persist in Europe [28, 29] and most characteristics identified by this systematic review are considered relevant in Albania.

### Study objective

The focus of this study is on childhood immunisation timeliness and vaccine confidence among mothers of under-5-year-old children in Albania. We analysed the 2017–2018 Albania Demographic and Health Survey (DHS) [30] to assess timeliness of childhood immunisation and vaccine confidence associated with health information source, maternal, child, socioeconomic, and geographic characteristics in Albania.

## Methods

### Survey data

Demographic and Health Surveys have been conducted in over 90 low- and middle-income countries [30]. The purpose of the 2017–18 Albania Demographic and Health survey was to obtain information on Albanian population’s sociodemographic and health indicators that can assist Albanian policymakers in the design and evaluation of health programs. Specifically, the survey was focused on fertility, family planning, nutrition, maternal and child health, knowledge of HIV behaviors, health-related lifestyle, and noncommunicable diseases.

For sampling, prefectures were stratified into urban and rural areas resulting in 24 sampling strata. A two-stage process was used to select enumeration areas (city blocks in urban areas and villages in rural areas). In the first stage, 715 EAs were selected with probability proportional to size and with independent selection in each sampling stratum. In the second stage, 24 households per enumeration area were sampled using an equal probability systematic selection. The resulting nationally representative sample included 17,160 households. The survey was conducted by trained field-workers between 11 September 2017 and 20 February 2018.

**Fig. 1:**
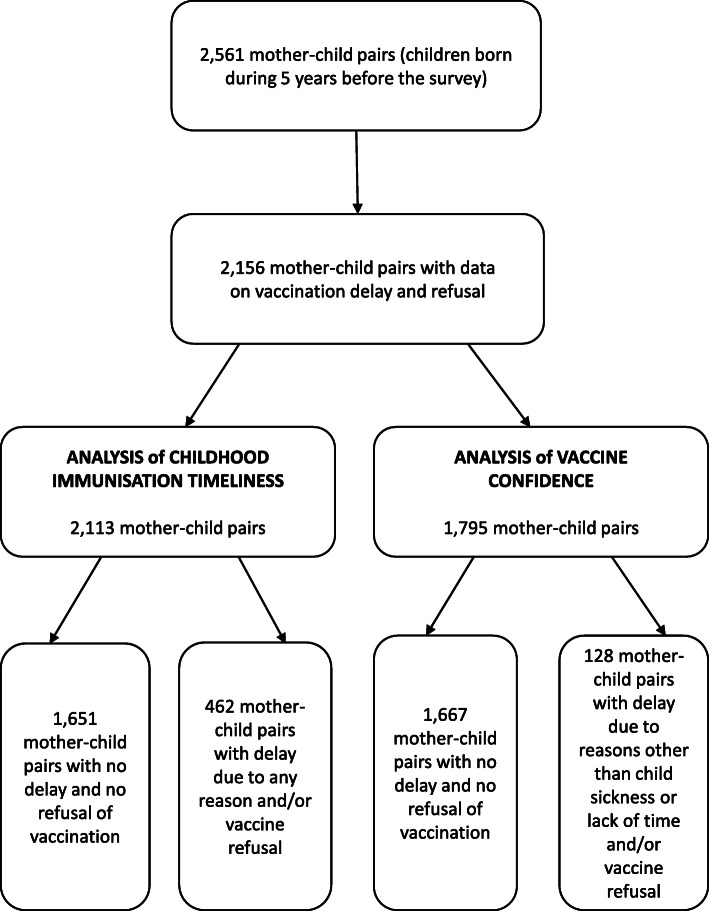
**Flow-chart illustrating subpopulations for childhood immunisation timeliness and vaccine confidence from the 2017–2018 Albania DHS.** Childhood immunisation timeliness refers to any delay and/or refusal of a vaccine and was analysed in a weighted sample of 2113 mother-child pairs. Vaccine confidence was analysed in a weighted sample of 1795 mother-child pairs (after exclusion of children vaccinated with delay due to sickness of the child or lack of time of the mother)

In this study, we used the 2017–2018 Albania Demographic and Health Survey data obtained via the questionnaire for women aged 15–49 years to estimate childhood immunisation timeliness and vaccine confidence [14]. The survey interview was completed by 10,860 women aged 15–49 years (93% of those eligible), and data were collected for a weighted sample of 2561 children born to these women within the last 5 years (before survey interview). We excluded data for 405 children due to missing data on vaccination delay and refusal, and we excluded an additional 13 observations with missing data on Internet/social media and 30 observations with missing data for the partner’s education and work. Thereby, we used sample weighted data for 2113 mother-child pairs in our analysis of immunisation timeliness. For the analysis of vaccine confidence, we excluded observations for children vaccinated with delay due to sickness (of the child) or lack of time of the mother, apart from observations with missing data on Internet/social media. Thereby, we used sample weighted data for 1795 mother-child pairs for our analysis of vaccine confidence, as shown in Fig. 1.

### Childhood immunisation timeliness and vaccine confidence analysis

The primary outcome variables are childhood immunisation timeliness and vaccine confidence.

We define childhood immunisation timeliness as no delaying and no refusing childhood immunisation, that is answers “no” to questions “Have you ever postponed or delayed having your child (one of your children) vaccinated?” and “Have you ever chosen not to have your child (one of your children) vaccinated?”. We define vaccine confidence as not refusing childhood immunisation or not delaying immunisation due to reasons other than sickness of the child or lack of time. The independent variables of interest were based on health information source, maternal, child, socioeconomic, and geographic characteristics. Health information source characteristics include Internet/social media as main source of health information and trusted sources of information on vaccines; maternal characteristics include age of childbirth, education, marital status, and household head status; child characteristics include gender and birth order; socioeconomic characteristics include mother’s work status, partner’s work status, partner’s education, household wealth, ethnicity, and religion; and geographic characteristics include area of residence (urban/rural) and prefecture.

We conducted simple logistic regression to estimate crude odds ratios and assess childhood immunisation timeliness and vaccine confidence disaggregated by the above mentioned characteristics. *P*-values were obtained by F-test. We followed up with multivariable logistic regression (N weighted = 2113 for immunisation timeliness, and N weighted = 1795 for vaccine confidence) to estimate the association of selected independent variables with immunisation timeliness and vaccine confidence after controlling for other background characteristics. Variables selected for the multivariable models were those associated with immunisation timeliness and vaccine confidence respectively in the univariate analyses, defined as with *p*-values < 0.05 and/or having a strong effect on immunisation timeliness and vaccine confidence respectively with odds ratio of OR ≤ 0.5 or OR ≥ 1.5.

The covariates selected for the multivariable model for immunisation timeliness were Internet/social media as the main health information source, trusted source of information on vaccines, maternal education, education of partner, mother’s work, partner’s work, ethnicity, religion, and prefecture. Marital status could not be analysed because there were no respondents in the category “other” following the exclusion of records with missing data on the partner’s education and work. Ethnicity and religion were excluded on the basis of multicollinearity with the region. The covariates selected for the multivariable model for vaccine confidence were Internet/social media as the main health information source, trusted source of information on vaccines, marital status, ethnicity, religion, household wealth, and prefecture. Religion and household wealth were excluded due to multicollinearity with region and Internet/social media as the main health information source respectively. In both analyses, the trusted source of information on vaccines was considered on the causal pathway between the Internet/social media and immunisation timeliness/vaccine confidence, and was therefore excluded. To avoid data sparsity, prefectures according to Eurostat NUTS3 division were grouped into NUTS2 regions [31] with minimal loss of precision. *P*-values were obtained by t-test.

### Ethics approval and reproducible analysis

This study was approved by the ethics committee (Ref 21362) of the London School of Hygiene & Tropical Medicine. The 2017–2018 Albania DHS dataset is accessible upon registration on the DHS website [30]. The survey analysis was conducted by taking into account stratification, cluster sampling, and sampling weights, using Stata statistical software [32], and visualisations were created using the R statistical software [33].  The code is accessible at https://github.com/d-mayerova/Vaccine_confidence_Albania. 

## Results

### Characteristics of the study population

Among mothers of under-5-year-old children in Albania, 30% of them reported the Internet as the main source of health information (see Table 1); other main sources were healthcare providers (42%), television (25%), friends/relatives (2.0%), schools (0.6%), newspapers (0.2%) and other (0.2%). Most women considered healthcare professionals as the most trusted source of information on vaccines. More than one-quarter of mothers completed higher education, while 1.2% did not receive any educatio n. Most respondents were Albanian, Muslim, married/living with a partner, and not working outside the home during the 12 months preceding the survey. Children born during the last 5 years before the survey were 51% males and 44% were first-born. Most households were headed by men at 85%. The proportion of partners of the participating women who did not receive any education was similar to women at 1.4%. The majority of partners (81%) had at least partially worked during the 12 months preceding the survey. The study population was relatively more urban (56%) than rural.

**Table 1:** **Childhood immunisation timeliness by health information source, maternal, child, socioeconomic, and geographic characteristics in Albania. **Childhood immunisation timeliness among mothers of under-5-year-old children by health information source, maternal, child, socioeconomic, and geographic characteristics in Albania. Crude odds ratios were estimated via simple logistic regression and adjusted odds ratios were estimated via multivariable logistic regression

Characteristics	Category	Mothers in each sub-group (N and %)	Immunisation timeliness (% and 95% CI)	Crude odds ratio		Adjusted odds ratio	
				OR a nd 95% CI	***p***-value (F-test)	AOR and 95% CI	***p***-value (t-test)
**Health information source**							
** Internet/social media as main health information source**	No	1472 (69.6)	80.3 (75.7, 84.2)	1	0.022	1	–
	Yes	641 (30.4)	73.2 (67.6, 78.2)	0.67 (0.48, 0.94)		**0.66 (0.47, 0.94)**	**0.020**
**Trusted source of information on vaccines**	Healthcare workers	2028 (95.9)	78.9 (75.1, 82.3)	1	0.139		
	Traditional media	31 (1.5)	60.8 (23.0, 89.0)	0.41 (0.08, 2.13)			
	Internet /social media	38 (1.8)	64.0 (39.1, 83.2)	0.48 (0.17, 1.36)			
	Family, other	16 (0.8)	48.4 (16.3, 81.9)	0.25 (0.05, 1.23)			
**Maternal**							
**Age at birth (years)**	< 25	685 (32.4)	79.9 (74.1, 84.6)	1	0.494		
	25–29	753 (35.6)	76.6 (71.3, 81.1)	0.82 (0.56, 1.20)			
	≥30	675 (32.0)	78.1 (72.5, 82.8)	0.90 (0.60, 1.33)			
**Education**	Higher	569 (26.9)	74.3 (67.4, 80.1)	1	0.032	1	–
	Secondary	521 (24.7)	81.5 (76.5, 85.7)	1.53 (0.99, 2.35)		0.94 (0.59, 1.52)	0.811
	Primary	998 (47.2)	79.3 (74.8, 83.2)	1.33 (0.92, 1.91)		0.64 (0.38, 1.05)	0.078
	No	25 (1.2)	48.0 (22.1, 75.1)	0.32 (0.10, 0.99)		**0.14 (0.03, 0.67)**	**0.014**
**Marital status**	Married/living with partner	2113 (100)	78.1 (74.3, 81.5)	NA	NA		
	Other - mother alone	0 (0)	NA	NA			
**Sex of household head**	Male	1799 (85.1)	78.4 (75.1, 81.5)	1	0.703		
	Female	314 (14.9)	76.3 (62.5, 86.1)	0.88 (0.46, 1.68)			
**Child**							
**Gender**	Male	1071 (50.7)	76.4 (71.9, 80.4)	1	0.114		
	Female	1042 (49.3)	79.9 (75.5, 83.7)	1.2 (0.95, 1.57)			
**Birth order**	First	921 (43.6)	7836 (74.2, 81.9)	1	0.246		
	Second	791 (37.5)	76.3 (71.3, 80.7)	0.89 (0.70, 1.13)			
	Third/more	401 (19.0)	81.3 (74.6, 85.5)	1.20 (0.81, 1.80)			
**Socioeconomic**							
**Ethnicity**	Albanian	2032 (96.2)	78.6 (74.7, 82.0)	1	0.089		
	Egyptian	40 (1.9)	78.7 (59.2, 90.4)	1.01 (0.39, 2.64)			
	Other (mostly Roma)	41 (1.9)	56.7 (34.6, 76.4)	0.36 (0.14, 0.90)			
**Religion**	Muslim	1763 (83.4)	77.7 (73.4, 81.5)	1	0.053		
	Catholic	210 (9.9)	86.2 (77.3, 92.0)	1.79 (0.94, 3.39)			
	Other	140 (6.6)	71.2 (61.2, 79.5)	0.71 (0.43, 1.16)			
**Mother worked last 12 months**	Not worked	1383 (65.4)	82.0 (78.5, 85.1)	1	0.0001	1	–
	Worked / partly worked	730 (34.6)	70.8 (64.2, 76.6)	0.53 (0.39, 0.72)		**0.65 (0.47, 0.91)**	**0.013**
**Household wealth**	Poorest	485 (22.9)	80.1 (74.7, 84.7)	1	0.471		
	Poorer	429 (20.3)	80.4 (75.2, 84.9)	1.02 (0.67, 1.55)			
	Middle	409 (19.4)	79.9 (72.7, 85.6)	0.99 (0.61, 1.60)			
	Richer	419 (19.8)	74.9 (67.4, 81.1)	0.74 (0.47, 1.16)			
	Richest	371 (17.6)	74.6 (64.8, 82.3)	0.73 (0.44, 1.21)			
**Education of partner**	Higher	344 (16.3)	68.6 (57.8, 77.7)	1	0.065	1	–
	Secondary	741 (35.1)	79.5 (74.7, 83.5)	1.77 (1.06, 2.95)		1.60 (0.92, 2.78)	0.093
	Primary	998 (47.2)	80.4 (75.8, 84.3)	1.87 (1.18, 2.98)		1.74 (0.93, 3.25)	0.081
	No	30 (1.4)	78.7 (60.2, 90.1)	1.69 (0.61, 4.69)		2.58 (0.39, 17.04)	0.324
**Partner worked last 12 months**	Not worked/don’t know	400 (18.8)	85.6 (79.7, 90.1)	1	0.012	1	–
	Worked/partly worked	1726 (81.2)	76.4 (72.0, 80.3)	0.54 (0.34, 0.88)		0.75 (0.45, 1.24)	0.263
**Geographic**							
**Area of residence**	Urban	1186 (56.1)	76.2 (69.8, 81.5)	1	0.158		
	Rural	927 (43.9)	80.7 (77.4, 83.6)	1.31 (0.90, 1.91)			
**Prefecture**	Tirane	560 (26.5)	71.1 (59.2, 80.6)	1	< 0.0001		
	Berat	102 (4.8)	68.5 (58.3, 77.2)	0.95 (0.49, 1.83)			
	Diber	124 (5.9)	93.6 (89.0, 96.4)	6.26 (2.97, 13.20)			
	Durres	241 (11.4)	86.8 (79.3, 91.8)	2.98 (1.45, 6.13)			
	Elbasan	238 (11.3)	75.9 (67.0, 83.0)	1.38 (0.72, 2.64)			
	Fier	225 (10.6)	70.5 (62.0, 77.8)	1.05 (0.57, 1.95)			
	Gjirokaster	42 (2.0)	62.3 (49.2, 73.9)	0.74 (0.36, 1.53)			
	Korce	154 (7.3)	70.2 (58.5, 79.7)	1.07 (0.53, 2.14)			
	Kukes	86 (4.1)	88.7 (81.0, 93.6)	3.61 (1.65, 7.89)			
	Lezhe	110 (5.2)	88.5 (78.2, 94.3)	3.40 (1.36, 8.49)			
	Shkoder	122 (5.8)	91.6 (83.6, 95.9)	4.97 (2.01, 12.25)			
	Vlore	109 (5.1)	90.6 (83.3, 94.9)	3.90 (1.78, 8.55)			
**Region**	AL01-Veri	684 (32.4)	89.4 (86.1, 92.0)	1	< 0.0001	1	
	AL02-Qender	798 (37.8)	72.5 (63.8, 79.8)	0.31 (0.19, 0.52)		**0.38 (0.23, 0.63)**	**< 0.0001**
	AL03-Jug	631 (29.9)	73.0 (68.4, 77.2)	0.32 (0.22, 0.47)		**0.36 (0.24, 0.53)**	**< 0.0001**

### Childhood immunisation timeliness

While 77.9% of mothers never delayed and never refused childhood immunisation, 21.3% of mothers reported immunisation delay, and 1.3% ever chose not to have their child/children vaccinated. Among mothers who delayed childhood vaccination, nearly two-thirds of them attributed the delay to the sickness of their child at the time of vaccination. Around 10% of the mothers attributed the delay due to lack of time while 11% of mothers indicated other reasons than those listed in the questionnaire (see Table 2). Around 6% of mothers stated vaccine safety and/or side effects were reasons for delaying vaccination, and an additional 6% of mothers stated no reason for immunisation delay. Among mothers who refused childhood immunisation, the main reasons were attributed to concerns about vaccine safety and side effects (see Table 2).

**Table 2:** Reasons for delaying childhood immunisation and refusing childhood immunisation in Albania

Reason	N	Proportion (% and 95 CI)
**Reasons stated by mothers of under-5-year-old children to delay childhood immunisation in Albania**		
Doubts about vaccine safety	12	2.8 (1.2, 6.1)
Concerns about side effects	15	3.3 (1.7, 6.0)
Child was sick at the time of vaccination	302	67.0 (59.3, 73.9)
Did not have time/too busy	43	9.6 (5.7, 15.8)
No particular reason	28	6.2 (3.7, 10.2)
Other	50	11.1 (7.5, 16.3)
**Reasons stated by mothers of under-5-year-old children to refuse childhood immunisation in Albania**		
Doubts about vaccine safety	13	47.8 (26.6, 69.8)
Concerns about side effects	6	23.10 (9.4, 46.7)
Child was sick at the time of vaccination	1	5.1 (0.1, 16.5)
Medical contraindication	0	0
Vaccine more harmful than disease	2	6.9 (1.5, 26.4)
Religious conviction	0	0
Other	5	17.1 (5.8, 40.6)

**Fig. 2:**
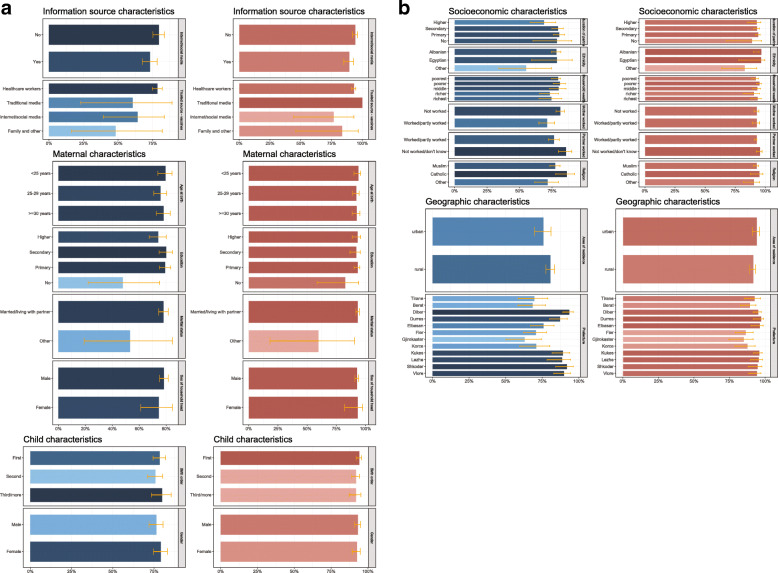
**Childhood immunisation timeliness and vaccine confidence among mothers in Albania**. Childhood immunisation timeliness (shown in blue colour) and vaccine confidence (shown in red colour) among mothers of under-5-year-old children in Albania disaggregated by health information source, child, and maternal characteristics (Fig. 2a), and disaggregated by socioeconomic, and geographic characteristics (Fig. 2b)

**Fig. 3:**
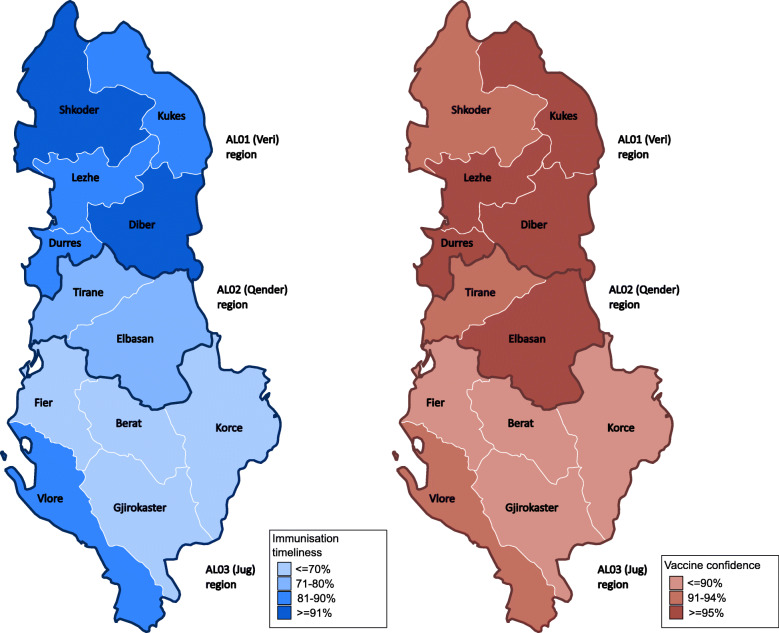
**Immunisation timeliness and vaccine confidence among mothers by prefectures and regions in Albania.** Immunisation timeliness and vaccine confidence among mothers of under-5-year-old children in childhood immunisation are higher in the AL01-Veri region, followed by AL02-Qender region and the AL03-Jug region

Childhood immunisation timeliness disaggregated by health information source, maternal, child, socioeconomic, and geographic characteristics in Albania are shown in Table 1 and Fig. 2. While immunisation timeliness was similar by urban and rural areas of residence, it was relatively high in the prefectures of AL01-Veri region (all > 80%, Shkoder and Diber > 90%), followed by prefectures (Elbasan of AL02-Qender region (71–80%), and relatively low in the prefectures of AL03-Jug region (< 70%) except for Vlore (see Fig. 3).

**Fig. 4:**
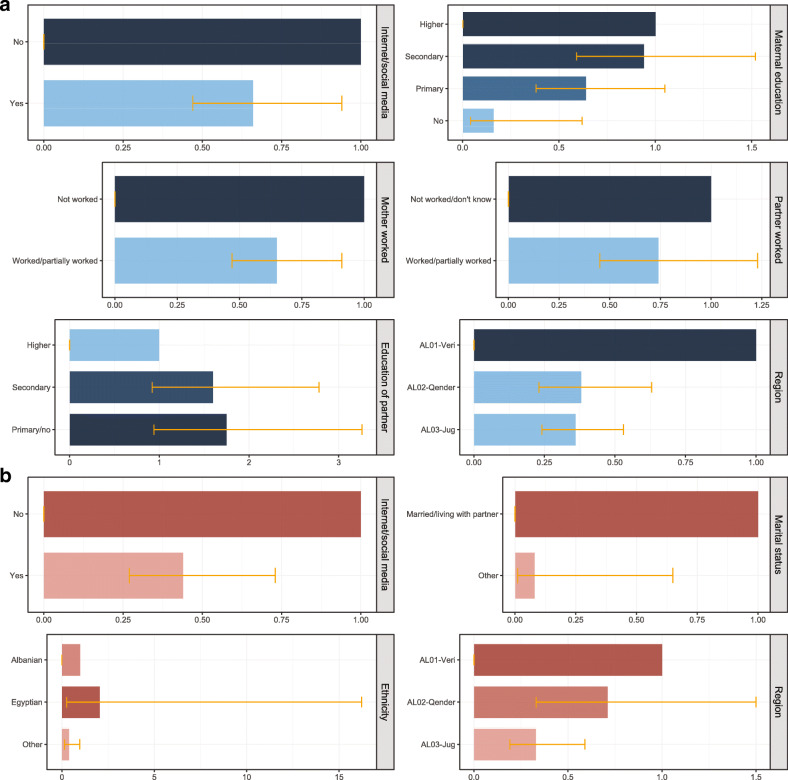
**Childhood immunisation timeliness and vaccine confidence in Albania, results from multivariable analyses.a** Childhood immunisation timeliness among mothers of under-5-year-old children associated with health information source (Internet/social media), maternal (education), socioeconomic (mother’s work, partner’s work, partner’s education), and geographic (region) characteristics in Albania. **b** Vaccine confidence among mothers of under-5-year-old children associated with health information source (Internet/social media), maternal (marital status), socioeconomic (ethnicity), and geographic (region) characteristics in Albania

Immunisation timeliness among mothers of under-5-year-old children associated with health information source (Internet/social media), maternal (education), socioeconomic (mother’s work, partner’s work, education of partner), and geographic (region) characteristics are illustrated in Table 1 and Fig. 4a. After controlling for other background characteristics in the multivariable logistic regression, mothers whose main health information source was the Internet/social media had 34% lower odds of childhood immunisation timeliness than mothers with other main information sources. Compared to non-working mothers, those who worked had 35% lower odds of immunisation timeliness. Mothers residing in AL02-Qender and AL03-Jug regions had 62 and 64% lower odds of childhood immunisation timeliness respectively in comparison to women living in AL01-Veri region. Mothers without education had 86% lower odds of immunisation timeliness compared to those who completed higher education; there was an association (*p* = 0.032) of decreasing immunisation timeliness with decreasing education level.

### Vaccine confidence

Vaccine confidence among the mothers was high at 92.9% and had similar geographical patterns to immunisation timeliness (see Fig. 3). After controlling for other background characteristics in the multivariable logistic regression, mothers using the Internet/social media as the main health information source had 56% lower odds of vaccine confidence compared to mothers who reported other main health information sources (see Table 3 and Fig. 4b). Single mothers (not married or living with a partner) had 92% lower odds of vaccine confidence compared to those married/living with a partner. Mothers from other ethnicities (mostly Roma) had 61% lower odds of vaccine confidence in comparison to mothers of Albanian ethnicity, and m others living in AL03-Jug region had 67% lower odds of vaccine confidence in comparison to mothers living in AL01-Veri region.

**Table 3:** Vaccine confidence by health information source, maternal, child, socioeconomic, and geographic characteristics in Albania. Vaccine confidence among mothers of under-5- year-old children by health information source, maternal, child, socioeconomic, and geographic characteristics in Albania. Crude odds ratios were estimated via simple logistic regression and adjusted odd s ratios were estimated via multivariable logistic regression

Characteristic	Category	Mothers in each sub-group (N and %)	Vaccine confidence (% and 95 CI)	Crude odds ratio		Adjusted odd ratio	
				OR (95% CI)	***p***-value (F-test)	AOR (95% CI)	***p***-value (t-test)
**Health information source **							
**Internet/social media as main health information source**	No	1268 (70.6)	94.3 (92.0, 95.9)	1	0.019	1	–
	Yes	527 (29.4)	89.5 (84.9, 92.8)	0.52 (0.30, 0.90)		**0.44 (0.27, 0.73)**	**0.002**
**Trusted source of information on vaccines**	Healthcare workers	1734 (96.6)	93.2 (91.2, 94.7)	1	0.093		
	Traditional media	19 (1.1)	100	–			
	Internet/ social media	32 (1.8)	76.7 (44.2, 93.2)	0.24 (0.06, 1.02)			
	Family, other	10 (0.5)	83.6 (45.9, 96.8)	0.37 (0.06, 2.27)			
**Maternal**							
**Age at birth (years)**	< 25	594 (33.1)	93.9 (90.9, 96.0)	1	0.604		
	25–29	627 (35.0)	92.3 (89.2, 94.6)	0.78 (0.47, 1.28)			
	≥30	574 (32.0)	92.6 (88.7, 95.2)	0.81 (0.42, 1.55)			
**Education**	Higher	454 (25.3)	93.2 (88.7, 96.0)	1	0.422		
	Secondary	464 (25.8)	92.3 (86.7, 95.6)	0.87 (0.38, 1.98)			
	Primary	862 (48.0)	93.3 (90.9, 95.1)	1.02 (0.53, 1.93)			
	No	15 (0.8)	82.7 (58.5, 94.2)	0.35 (0.09, 23.98)			
**Marital status**	Married/living with partner	1768 (98.5)	93.4 (91.8, 94.8)	1	0.022	1	–
	Other - mother alone	27 (1.5)	59.7 (18.1, 90.8)	0.10 (0.02, 0.72)		**0.08 (0.01, 0.65)**	**0.019**
**Sex of household head**	Male	1529 (85.2)	92.8 (91.0, 94.4)	1	0.889		
	Female	911 (14.8)	93.4 (82.1, 97.7)	1.08 (0.34, 3.46)			
**Child**							
**Gender**	Male	884 (42.3)	93.3 (90.9, 95.0)	1	0.697		
	Female	911 (50.7)	92.6 (89.3, 94.9)	0.90 (0.54, 1.50)			
**Birth order**	First	779 (43.4)	94.1 (92.0, 95.6)	1	0.324		
	Second	661 (36.8)	92.0 (88.9, 94.3)	0.72 (0.47, 1.11)			
	Third/more	355 (19.8)	92.1 (87.3, 95.2)	0.73 (0.40, 1.36)			
**Socioeconomic**							
**Ethnicity**	Albanian	1734 (96.6)	93.0 (91.0, 94.6)	1	0.133	1	–
	Egyptian	33 (1.8)	96.4 (77.8, 99.5)	2.00 (0.26, 15.40)		2.05 (0.26, 16.22)	0.496
	Other	28 (1.6)	82.8 (63.7, 93.0)	0.36 (0.13, 1.05)		**0.39 (0.15, 0.97)**	**0.042**
**Religion**	Muslim	1492 (83.1)	92.8 (90.1, 94.5)	1	0.532		
	Catholic	191 (10.6)	95.1 (87.4, 98.1)	1.50 (0.52, 4.37)			
	Other	112 (6.2)	90.4 (81.8, 95.2)	0.73 (0.33, 1.62)			
**Mother worked last 12 months**	Not worked	1228 (68.4)	92.9 (90.5, 94.7)	1	0.986		
	Worked/partly worked	567 (31.6)	92.9 (89.0, 95.5)	1.00 (0.56, 1.80)			
**Household wealth**	Poorest	427 (23.8)	91.9 (88.5, 94.4)	1	0.376		
	Poore	372 (20.8)	95.2 (92.5, 97.0)	1.75 (0.97, 3.12)			
	Middle	352 (19.6)	93.5 (89.4, 96.1)	1.26 (0.64, 2.47)			
	Richer	348 (19.4)	90.1 (82.0, 95.3)	0.84 (0.36, 1.97)			
	Richest	296 (16.5)	93.5 (87.8, 96.7)	1.27 (0.57, 2.84)			
**Education of partner**	Higher	256 (14.5)	92.5 (85.4, 96.3)	1	0.756		
	Secondary	633 (35.8)	93.1 (90.2, 95.2)	1.09 (0.46, 2.59)			
	Primary	854 (48.3)	94.0 (91.7, 95.7)	1.27 (0.56, 2.91)			
	No	26 (1.5)	88.9 (67.3, 96.9)	0.65 (0.14, 3.11)			
**Partner worked last 12 months**	Not worked/don’t know	356 (20.1)	95.6 (92.6, 97.4)	1	0.117		
	Worked/partly worked	1413 (79.9)	92.9 (90.9, 94.5)	0.61 (0.33, 1.13)			
**Geographic**							
**Area of residence**	Urban	971 (54.1)	94.0 (90.9, 96.1)	1	0.196		
	Rural	824 (45.9)	91.7 (89.1, 93.7)	0.70 (0.41, 1.21)			
**Prefecture**	Tirane	430 (24.0)	92.5 (85.3, 96.4)	1	0.004		
	Berat	78 (4.4)	89.1 (82.3, 93.5)	0.66 (0.26, 1.70)			
	Diber	123 (6.9)	95.1 (91.4, 97.3)	1.58 (0.60, 4.17)			
	Durres	221 (12.3)	97.0 (91.6, 98.9)	2.59 (0.69, 9.69)			
	Elbasan	188 (10.5)	96.1 (89.9, 98.6)	2.01 (0.56, 7.22)			
	Fier	185 (10.3)	86.4 (78.8, 91.6)	0.51 (0.20, 1.31)			
	Gjirokaster	32 (1.8)	84.9 (74.3, 91.6)	0.46 (0.17, 1.25)			
	Korce	127 (7.1)	87.4 (78.7, 92.9)	0.56 (0.21, 1.51)			
	Kukes	82 (4.6)	95.8 (91.7, 97.9)	1.82 (0.64, 5.18)			
	Lezhe	102 (5.7)	95.4 (89.5, 98.0)	1.68 (0.52, 5.42)			
	Shkoder	121 (6.7)	94.6 (87.9, 97.7)	1.41 (0.44, 4.51)			
	Vlore	106 (5.9)	94.1 (88.3, 97.2)	1.30 (0.44, 3.80)			
**Region**	AL01-Veri	649 (36.1)	95.8 (93.7, 97.2)	1	0.0002	1	–
	AL02-Qender	618 (34.4)	93.6 (88.4, 96.5)	0.65 (0.30, 1.40)		0.71 (0.33, 1.50)	0.367
	AL03-Jug	528 (29.4)	88.5 (85.0, 91.3)	0.34 (0.20, 0.57)		**0.33 (0.19, 0.59)**	**< 0.0001**

## Discussion

We examined most of the sociodemographic characteristics identified by systematic reviews as risk factors for delays in childhood immunisation across countries [6, 34]. This is the first study, to our knowledge, to assess childhood immunisation timeliness and vaccine confidence associated with health information source, maternal, child, socioeconomic, and geographic characteristics in Albania.

### Main findings and comparison with other studies

We estimated that more than three-quarters of mothers adhered to childhood immunisation schedules in Albania. Around one in five mothers reported immunisation delay, and one in nearly 80 mothers ever refused a vaccine. Delays in childhood immunisation were primarily due to the sickness of the child at the time of vaccination, while other reasons included lack of time, doubts about vaccine safety, and concerns about side effects. Concerns about vaccine safety and side effects were the most frequently reported reason for vaccine refusal. Lower immunisation timeliness was associated with having the Internet/social media as the main health information source in comparison to other sources, mother’s work outside the home compared to being in a household, and living in AL02-Qender and AL03-Jug regions in comparison to AL01-Veri region. Further, mothers without education had lower odds of childhood immunisation timeliness compared to those who completed higher education ; however, the sample size was small. More than nine mothers in ten had confidence in childhood immunisation. Lower vaccine confidence was associated with having the Internet/social media as the main health information source in comparison to other sources, not being married or living with partner, certain ethnic minorities (like Roma) in comparison to Albanian ethnicity, and living in AL03-Jug region in comparison to AL01-Veri region.

In regard to related studies, a survey about immunisation concerns among Albanian parents reported higher immunisation timeliness, lower rates of delay, and higher vaccine refusal rates [15]. Our estimate of delays in childhood immunisation in Albania is similar to other countries of the Balcan peninsula, Croatia [35], and the neighbouring country of Greece for traditional vaccines (timeliness at 12 and 24 months) [36]. We inferred a relatively lower vaccine rejection rate in comparison to findings from a survey on vaccine confidence among 18 European countries [9] and the Croatian [35] survey. Data obtained from immunisation registries for a sub-sample of children of the interviewed mothers – 925 children up to 35 months old – revealed a higher immunisation delay of 29% and refusal of 19% (missing one or more basic vaccinations) [14]. However, the registry-based data did not include older 3-year and 4-year old children whose immunisation might be more compliant with schedules compared to infants up to 35 months old, supporting the DHS finding of increasing immunisation delay and/or vaccine refusal rates. Also, other reasons may contribute to this difference, including poor memory recall or social desirability bias leading to under-reporting of alternative immunisation schedules, and disproportionately high non-response to questions on immunisation timeliness among mothers who delay and/or refuse childhood vaccination.

The time-frame for vaccination delay was not defined in the questionnaire. This may result in over-reporting of the delay compared to studies where vaccine timeliness was obtained from immunisation records and checked against a predefined time-frame. However, the opposite effect is also possible, depending on the chosen time-frame (in the absence of an internationally agreed definition of vaccine timeliness [6]).

Our finding that the main reasons for childhood immunisation delay and/or refusal are due to adverse circumstances of child sickness and mothers being too busy rather than an intention is consistent with the literature [9, 37, 38]. The survey examining vaccine concerns in Albania [15] did not examine delay and refusal separately, but concerns about safety and adverse effects were the most frequently reported. Overall, doubts about vaccine safety belong to the most critical determinants of vaccine confidence in Europe [4, 5]. Further, 11% of women who delayed or refused immunisation had other reasons than those specified in the questionnaire. Two studies among Albanian caregivers identified additional concerns that might have possibly contributed to the “other” reason category – too many vaccines given to infants [15, 39], distrust in vaccines/effectiveness of vaccines provided by the state, and lack of information about vaccination [15]. The vaccine confidence survey of 18 European countries estimated that 88 and 80% of parents have not refused and have not delayed childhood immunisation for reasons other than illness or allergy, respectively [9]. This measure is similar to our vaccine confidence estimate for Albania.

We inferred lower childhood immunisation timeliness and lower vaccine confidence (higher hesitancy) among mothers in Albania who mainly used the Internet/social media for health information, similar to findings in France [24]. In contrast, studies from Poland [40] and the United States [41] found no association between the Internet as a vaccine information source and alternative childhood vaccination schedules. Further, other studies have indicated an adverse impact of the Internet on childhood immunisation, though not through the same relationship of Internet versus immunisation timeliness and vaccine confidence but with regard to the role of the Internet on vaccination decisions [26] and perceptions on vaccine safety and effectiveness [42].

We inferred lower childhood immunisation timeliness among mothers who work outside their homes in Albania, and this was consistent with the findings of a systematic review [34] and analysis of DHS surveys from 96 countries [43]. Mothers working outside the home may encounter more difficulties when planning immunisation visits than mothers in households; however, lack of time was reported only by less than one-tenth of the respondents who delayed childhood vaccination. Further in our study, maternal work status was not associated with vaccine confidence either in Albania. Being a single mother has been a common predictor of poorer immunisation timeliness/coverage across countries [34, 43], and we also inferred relatively lower vaccine confidence among them in comparison to married mothers or living with a partner. This may indicate other factors beyond time constraints that influence childhood immunisation decisions among single mothers in Albania, although the sample size was small. Lower vaccine confidence observed among mothers of certain ethnic minorities like Roma may contribute to lower immunisation coverage of Roma children observed in Albania and other countries [13, 44], however, the sample size was small.

We inferred lower adherence to immunisation timelines among mothers residing in AL02-Qender and AL03-Jug regions in comparison to mothers in to AL01-Veri region, and lower vaccine confidence among mothers in AL03-Jug region in comparison to AL01-Veri region, though the reasons are unclear. With regard to education, there is conflicting evidence on the association of parental education with childhood immunisation timelines and vaccin e confidence [5, 34, 36, 45, 46]. 

### Limitations 

We acknowledge our study limitations. We did not analyse childhood immunisation timeliness and vaccine confidence for individual vaccines. We did not consider other factors that may contribute to delaying or refusing childho od vaccines such as constrai nts to vaccine supply, access and health workforce [6, 47]. Childhood immunisation delay and refusal were based on maternal reporting which was subject to information (recall) bias and poor memory recall that can lead to misclassification. Poor memory recall or non-differential misclassification could lead to under-estimation of the observed associations. Differential misclassification would distort the strength of the associations also towards the null since mothers who do not adhere to immunisation schedules might feel uncomfortable with questions on vaccines and mig ht not provide accurate answers (social desirability bias). The work status of parents during the last 12 months was a proxy measure of their work at the time of childhood immunisation, which may result in residual confounding.

### Future directions

Our recommendations for further research include prospective design, retrieving immunisation data for individual vaccines from registries, defining a time-frame for childhood immunisation timeliness, and a larger sample size to better identify the factors for declining immunisation timeliness and coverage among the Albanian children.

## Conclusions

In conclusion, our analysis of the 2017–2018 Albania Demographic and Health Survey indicates an inverse relationship between the Internet/social media as the main health information source and childhood immunisation timeliness and vaccine confidence in Albania. We inferred that lower immunisation timeliness was associated with using the Internet/social media as the main health information source compared to other sources, mother’s work outside the home, lack of maternal education, and living in AL02-Qender and AL03-Jug regions in comparison to AL01-Veri region. While we inferred that overall vaccine confidence was high in Albania, vaccine confidence was relatively lower among mothers using Internet/social media as the main health information source compared to other sources, not married or living with a partner, specific minority ethnicities such as Roma, and living in AL03-Jug region. The public health implications are that reinforcement of scientific evidence-based online communication about childhood immunisation in combination with tracking and analysis of vaccine hesitancy sentiment and anti-vaccination movements on the Internet/social media would be beneficial in improving immunisation timeliness and vaccine confidence in Albania. Since parents tend to search online for information that would confirm their original beliefs [48], traditional ways of promoting vaccination by healthcare professionals who enjoy confidence as trusted sources of health information should be sustained and strengthened to target the inequities in childhood immunisation timelines and vaccine confidence in Albania.

## Data Availability

The analysis code is publicly accessible at https://github.com/d-mayerova/Vaccine_confidence_Albania and the 2017–2018 Albania DHS data set is accessible upon registration on the DHS website at https://dhsprogram.com/methodology/survey/survey-display-525.cfm. To download DHS datasets, researchers must register as a DHS data user at https://dhsprogram.com/data/new-user-registration.cfm.

## Abbreviations

AOR: Adjusted odds ratio
BCG: Bacillus Calmette-Guérin
DHS: Demographic and Health Survey
DPT-HepB-Hib: Diphtheria/pertussis/tetanus/hepatitis B/*Haemophilus influenzae* type b
DTP3: Third dose of diphtheria-tetanus-pertussis vaccine
HIV/AIDS: Human immunodeficiency virus/Acquired immunodeficiency syndrome
MMR: Measles-mumps-rubella vaccine
NUTS: Nomenclature of territorial units for statistics
OR: Odds ratio
PCV: Pneumococcal conjugate vaccine
WHO: World Health Organization
95% CI: 95% confidence interval
